# Smoking in context – a multilevel approach to smoking among females in Helsinki

**DOI:** 10.1186/1471-2458-8-134

**Published:** 2008-04-24

**Authors:** Sakari Karvonen, Petteri Sipilä, Pekka Martikainen, Ossi Rahkonen, Mikko Laaksonen

**Affiliations:** 1 STAKES, National Research and Development Centre for Welfare and Health, Box 220, 00531 Helsinki, Finland; 2 Department of Sociology, Box 18, 00014 University of Helsinki, Finland; 3 Department of Public Health, Box 41, 00014 University of Helsinki, Finland

## Abstract

**Background:**

Smoking is associated with disadvantage. As people with lower social status reside in less privileged areas, the extent of contextual influences for smoking remains unclear. The aims were to examine the spatial patterning of daily smoking within the city of Helsinki, to analyse whether contextual variation can be observed and which spatial factors associate with current daily smoking in the employed female population.

**Methods:**

Data from a cross-sectional questionnaire were collected for municipal employees of Helsinki (aged 40–60 years). The response rate was 69%. As almost 4/5 of the employees are females, the analyses were restricted to women (n = 5028). Measures included smoking status, individual level socio-demographic characteristics (age, occupational social class, education, family type) and statistical data describing areas in terms of social structure (unemployment rate, proportion of manual workers) and social cohesion (proportions of single parents and single households). Logistic multilevel analysis was used to analyse data.

**Results:**

After adjusting for the individual-level composition, smoking was significantly more prevalent according to all social structural and social cohesion indicators apart from the proportion of manual workers. For example, high unemployment in the area of domicile increased the risk of smoking by almost a half. The largest observed area difference in smoking – 8 percentage points – was found according to the proportion of single households.

**Conclusion:**

The large variation in smoking rates between areas appears mainly to result from variation in the characteristics of residents within areas. Yet, living in an area with a high level of unemployment appears to be an additional risk for smoking that cannot be fully accounted for by individual level characteristics even in a cohort of female municipal employees.

## Background

Smoking is increasingly associated with many markers of deprivation at the individual level. Such markers include single parenthood, material poverty, poor socioeconomic status (education, occupation, employment) and poor housing conditions [1-3]. Smoking cessation has also been shown to be inversely related to socio-economic position [4,5]. Contextual factors have recently become a focus of research into the social determinants of health [6-9]. This research suggests that smoking is also influenced by collective factors such as neighbourhood of residence [10,11], workplace [12] and school environment among young people [13,14].

Regional differences in smoking have been well described [15-20]. Studies applying contemporary methodology on smoking among adults suggest, however, that for example in the UK major part of differences in smoking between regions is accounted for by the social composition of the areas [21]. In contrast, in Scotland Hart et al. [22] found no association between area and smoking after adjusting for the social composition of the residents between the districts [also [23,24]]. Ecob and Macintyre [25], on the other hand reported current smoking to be associated with deprivation in the areas in the West of Scotland, although age and household deprivation modified the association. On the whole, most studies suggest a moderate positive association between area deprivation and smoking after controlling for individual-level social composition [26-35].

An Indian study found significant contextual variation in smoking according to local area, district and even state [36]. Indeed, given the plethora of smoking measures applied (e.g. frequency, intensity, cessation), range (from neighbourhoods to states) and type of area indicators (e.g. crime rate, the extent of social cohesion, poverty and income indicators), earlier results present a quite mixed picture on the potential pathways to explaining how and by which mechanisms smoking is influenced by the small area context. An ideal spatial context for an exploration of smoking patterns by small area would comprise a reasonably stable and homogeneous population with relatively low variation of disadvantage. As smoking concentrates increasingly among the most disadvantaged – typically unemployed men – studies reporting contextual effects on smoking can run the risk of reflecting processes taking place predominantly among the least advantaged. To avoid the problems inherent in modifiable area units it is also essential that the context is clearly defined in terms of geography in order to represent meaningful neighbourhoods.

To take into account the fact that the area effects may be sensitive to the context (e.g. urban-rural), population group (male-female, employed-unemployed) or area unit (large cultural or administrative area units vs. locally meaningful units) we have limited our study to females employed by the City of Helsinki and residing within Helsinki, the capital of Finland. Due to active city planning and housing policies Helsinki can be spatially characterised with a relatively small degree of gentrification but in terms of reputation, ethnicity and socio-economic distribution, neighbourhoods can be clearly distinguished [37].

By choosing a more limited focus we aim to assess whether the contextual effects suggested by earlier studies result mainly from patterns typical of the less privileged population groups. For example, as poverty and unemployment are likely to hinder a person's options for daily life due to more limited mobility; they may also be more subject to the influence of characteristics of localities. Further, earlier studies suggest that contextual patterns of smoking may differ by gender, even though research focussing specifically on females is rare [26].

The general aim of this study was to investigate whether the association between smoking and deprivation of the small local area can be found among employed females as well. A failure to establish the relationship would contribute to specifying the mechanisms through which spatial influences may operate.

Specifically, we aim, first, to explore whether smoking varies by the area of residence, and, second, to find out the extent to which this variation results from the composition of the population in the area. Third, we examine whether the social and economic characteristics of the area – particularly indicators of social structure (proportion of unemployed and of manual workers) and cohesion (proportion of single parents and of single households) can explain smoking in the target population.

Currently, the theoretical understanding of the mechanisms explaining potential associations between area characteristics and smoking behaviour is limited. We assume that the main process connecting the social structure of the area with individuals' smoking operates via contagion [16,38]. In other words, in areas with high rates of smoking among the unemployed or manual worker population, smoking behaviour disseminates more quickly and also spreads more easily to those population groups that are otherwise less likely to smoke (e.g. females). While the unemployment rate and the proportion of manual workers reflects the social structure of the area, social cohesion, on the other hand, is reflected in the quality of the social life. In this way social cohesion may be hypothesised as influencing smoking behaviour more indirectly [39]. Areas with a high rate of single households are assumed to be characterised with a shallower stratum of social contacts than areas that have lower rates. Not only do families provide social and emotional support for their members but also their social life tends to be more established. Single-parent households, in turn, may lack other resources (e.g. time) necessary to lead a socially active life which may affect the quality of their sociability. A low rate of single parents or of single households are thus hypothesised to create a normative milieu protecting also from practices that are (at the level of the society) perceived as undesirable, such as smoking.

## Methods

### Data

The data were derived from the Helsinki Health Study [40], which examines socioeconomic and other determinants of health and well-being among middle-aged men and women employed by the City of Helsinki. The City of Helsinki is the largest employer in Finland with nearly 40,000 employees. In addition to general administration, most people work in social and health care, education and culture, public transport, and in the technical and construction sectors.

We used cross-sectional baseline data that were gathered in two separate surveys in 2000 and 2001. Employer records were used to identify all employees of the City of Helsinki. A self-administered questionnaire was sent to all employees who reached the age of 40, 45, 50, 55 or 60 in the year of the survey. The overall response rate among women was 69% in both years. The total number of respondents was 6243, of whom 80% (5028) were women, which is why the analyses were restricted to women. The non-response analysis showed that respondents correspond to the target population reasonably well, except for younger respondents and manual workers being slightly underrepresented [41]. The questionnaires were sent to the employees' workplace. Home addresses are not easily available for non-respondents, and we can thus not identify their area of residence. Previous non-response analyses show that differences in response rate by various socio-demographic background variables were small [42,43]. This suggests that major differences in response rates by area are unlikely. To decrease the influence of possible area-variation in response, small areas were grouped into quartiles according to each area-level variable. Albeit the full information of individual areas is used in the models, our main interest focuses into the fixed part of the models in which area effects are manifested in these area quartiles.

### Measures

Smoking status was inquired with the question "Do you smoke cigarettes, cigars or a pipe daily?" with just two response alternatives, yes and no. One-fifth of women were daily smokers. Smokers were almost exclusively cigarette smokers, with only about 0.1% of women smoking a pipe or cigars.

Individual level socio-demographic characteristics were age, occupational class, educational attainment, and family type. Age was obtained from register-based information for the sample frame, whereas other measures were based on self-reports. Educational attainment was measured by a question asking about completed general or vocational education. Education was divided into four groups that correspond to elementary (compulsory) education (less than 10 years, 20% of respondents), vocational education (21%), secondary education (10–12 years, 34%) and higher education (university degree) (more than 12 years, 25%).

Occupational class consisted of four hierarchical groups: managers and professionals, semi-professionals, routine non-manuals, and manual workers. Manual workers and non-manual employees were separated using the socioeconomic classification of Statistics Finland and non-manual employees were further divided into four groups according to the occupational classification of the City of Helsinki. The routine non-manual class was the largest (43%) whereas only 11% came from the manual class. There were few female managers and they were merged with professionals (together 27%). The semi-professionals accounted for 19% of women.

Family type was constructed using information on marital status and having dependent children in the household. Family type was categorised into four groups: single (not married, no children) (19%), single parent (8%), married, no dependent children (37%) and married with dependent children (36%).

Contextual measures were retrieved from published official (City Office of Helsinki, Helsinki Region Statistics) statistics describing the 107 neighbourhoods of Helsinki. These areas are of the size that most residents could walk across them in 15–20 minutes and have an average population of 4000. Area-level indicators measured the social structure of the area (unemployment rate and the proportion of manual workers) and social cohesion (proportions of single and single-parent households). These variables were divided into quartiles for the analyses (Table 1).

**Table 1:** Distribution of the contextual indicators in neighbourhood quartiles, the prevalence of daily smoking [95% c.i.] and the distribution of study population in the corresponding quartiles

Indicator	Quartile 1	Quartile 2	Quartile 3	Quartile 4	All
% Unemployed (1)					
mean	3.7	6.3	8.3	12.3	7.7
min-max	0.0–5.1	5.2–7.3	7.5–10.0	10.1–15.9	0.0–15.9
% daily smokers	18.5 [15.6–21.5]	20.3 [17.9–22.8]	25.7 [23.2–28.1]	23.9 [21.5–26.4]	22.7 [21.4–24.0]
n (%)	669 (16.3)	1013 (24.6)	1224 (29.8)	1207 (29.3)	4113 (100)
% Manual workers (2)					
mean	14.1	20.6	27.8	39.8	26.4
min-max	6.8–18.6	18.7–25.3	26.3–30.9	31.0–100.0	6.8–100.0
% daily smokers	21.6 [18.7–24.5]	19.3 [16.9–21.7]	23.6 [21.4–25.7]	26.1 [23.2–29.0]	22.7 [21.4–24.0]
n (%)	770 (18.7)	1010 (24.6)	1464 (35.6)	869 (21.1)	4113 (100)
% Single households (3)					
mean	21.0	35.6	47.7	58.4	41.5
min-max	8.7–30.4	30.5–42.6	42.7–52.9	53.3–74.5	8.7–74.5
% daily smokers	17.4 [14.9–20.0]	23.0 [20.6–25.5]	25.7 [23.4–28.1]	22.8 [20.0–25.6]	22.7 [21.4–24.0]
n (%)	850 (20.7)	1115 (27.1)	1298 (31.6)	850 (20.7)	4113 (100)
% Single-parent families(4)					
mean	7.9	13.4	17.7	23.1	15.9
min-max	0.0–11.7	11.8–15.4	15.7–20.5	20.7–27.9	0.0–27.9
% daily smokers	19.2 [16.2–22.2]	20.5 [17.8–23.2]	25.1 [22.7–27.4]	23.5 [21.2–25.8]	22.7 [21.4–24.0]
n (%)	663 (16.1)	834 (20.3)	1296 (32.0)	1320 (32.1)	4113 (100)

(1) % employed of total labour force. Year 2001. Source: City of Helsinki Urban Facts.

(2) % manual workers of economically active residents. Year 2000. Source: Helsinki Region Statistics.

(3) % single households of all households. Year 2001. Helsinki Region Statistics

(4) % single-parent families of all families. Year 2001. Helsinki Region Statistics

### Statistical methods

First, the distribution of area indicators (mean, minimum and maximum) for 107 neighbourhoods in Helsinki was calculated in quartiles. The prevalence of daily smoking with 95% confidence intervals was also calculated in the corresponding quartiles of area indicators to assess the extent of area variation in smoking. Second, smoking prevalence was studied by the individual characteristics of social background by means of cross-tabulation and Pearson's χ^2^.

The modelling part of the analysis was conducted using the GLIMMIX macro of SAS [44]. The estimates yielded by the GLIMMIX macro are regarded as an approximation of the maximum likelihood (ML) method. Our choice of estimation method is produced merely by a requirement for corrected estimates for the fixed part of the models (regression coefficients and standard errors of the area level covariates), as we do not report any estimates of variance at the area-level in the tables.

In the main stage of analysis, two-level logistic regression models were fitted stepwise. In the base model only the unadjusted neighbourhood variance was estimated. Age was controlled for in the first model, with family type in the second model, and socioeconomic status indicators (educational attainment and occupational class) in the third model. The results of these analyses are presented by showing area effects (odds ratios and their 95% confidence intervals) for daily smoking according to quartiles of area indicators in each of the three models. The area indicators were included separately, because a model combining all four area indicators would have suffered from multicollinearity.

## Results

Substantial differences existed between the neighbourhoods in terms of social structure and cohesion (Table 1). In the highest neighbourhood quartile, the unemployment rate was more than three-fold compared with the lowest neighbourhood quartile. Respectively, there was almost a three-fold difference in the proportion of manual workers, single households and single-parent families between the highest and the lowest neighbourhood quartiles.

The variation in daily smoking by neighbourhood was weaker than the variation in the area indicators. Furthermore, daily smoking was patterned by area so that it was most prevalent in the second most deprived quartile according to all other indicators except for the proportion of manual workers. The least deprived areas consistently showed the lowest rates of smoking in the three other area indicators (%unemployed, %single households and %single parents).

The largest observed area difference in smoking – of 8.3 percentage points – was between the first and the third quartile of the proportion of single households. Smoking was also statistically significantly more prevalent in the third unemployment quartile than in the lowest and the second lowest quartiles. Similarly, according to single households and single parents, smoking was more frequent in the third quartile than in the lowest quartile. In contrast, smoking was more prevalent in the highest quartile of manual workers (the most deprived areas) than in the second quartile.

Smoking was likewise associated with all individual-level sociodemographic characteristics (Table 2). Smoking was most prevalent among the second youngest age group (45 years) and lowest among the oldest age group (60 years). Smoking increased with decreasing educational level and with occupational class so that smoking was more than 2.5 times more common both for the least educated compared to the highest educated and for the lowest occupational class group compared to the highest occupational class group. The highest frequency of smoking was among single mothers but the differences between other family types were not statistically significant.

**Table 2:** Prevalence of daily smoking [95% c.i.] by individual characteristics and the distribution of study population over the categories

Variable	Prevalence of daily smoking [95% C.I.]	N (%)	p-value (Pearson's χ^2^)
Age			< 0.0001
40	22.5 [19.6–25.4]	822 (20.0)	
45	29.3 [26.3–32.3]	897 (21.8)	
50	25.4 [22.6–28.2]	913 (22.2)	
55	19.5 [17.1–22.1]	989 (24.0)	
60	12.2 [9.3–15.1]	492 (12.0)	
Education			< 0.0001
elementary	33.8 [30.6–37.0]	841 (20.4)	
vocational	27.2 [24.3–30.1]	880 (21.4)	
secondary	20.6 [18.5–22.7]	1377 (33.5)	
university degree	12.4 [10.4–14.4]	1015 (24.7)	
Occupational SES			< 0.0001
manual	34.6 [30.3–38.9]	463 (11.3)	
routine non-manual	27.9 [25.8–30.0]	1759 (42.8)	
semi-professional	17.1 [14.5–19.7]	776 (18.9)	
managerial/professional	13.5 [11.4–15.4]	1115 (27.1)	
Family type			< 0.0001
single	23.2 [20.2–26.2]	775 (18.8)	
single parent	32.4 [27.2–37.4]	327 (8.0)	
2 or more adults + no children	22.9 [20.8–25.0]	1544 (37.5)	
2 or more adults + children	20.0 [18.0–22.0]	1467 (35.7)	
ALL	22.7 [21.4–24.0]	4113 (100)	

In sum, the cross-tabulations showed that daily smoking varies both between individual level characteristics and between the areas. The area differences were, however, clearly smaller than those between individual characteristics, particularly age, educational and occupational class groups. Correlations between the area indicators were low (between 0.1 and 0.2) apart from the correlation between unemployment and manual workers (r = 0.5) and the correlation between single households and single-parent households (r = -0.5).

Area-level differences were further studied by a two-level logistic regression analysis. The area-level variance was not statistically significant (0.03756, p = 0.0622) even in the unadjusted base model. Every subsequent adjustment attenuated the variance further. We thus do not report the variances in the tables, but concentrate on the regression coefficients of the fixed part of the models.

After adjusting for the individual level characteristics (Table 3) the area-level indicators remained associated with smoking similarly to the bivariate analysis (Table 1). After adjusting for all four individual-level characteristics, smoking was elevated in the areas of the third highest rate of unemployment and of the third highest proportion of single-parent families. Furthermore, smoking was less prevalent in the areas with the lowest rate of single households than in the other areas.

**Table 3:** Associations of the area-level indicators with daily smoking in models adjusted for the individual characteristics (107 areas) [Odds ratio and 95 % c.i.]

Indicator	Model 1	Model 2	Model 3
% Unemployed^1^			
Lowest quartile	1.00	1.00	1.00
2^nd ^quartile	1.14 [0.88–1.47]	1.15 [0.89–1.49]	1.11 [0.85–1.46]
3^rd ^quartile	1.54 [1.21–1.96]	1.54 [1.21–1.97]	1.45 [1.12–1.88]
Highest quartile	1.41 [1.10–1.80]	1.38 [1.08–1.77]	1.28 [0.98–1.65]
% Manual workers^2^			
Lowest quartile	1.00	1.00	1.00
2^nd ^quartile	0.86 [0.67–1.11]	0.87 [0.68–1.13]	0.83 [0.64–1.08]
3^rd ^quartile	1.12 [0.89–1.41]	1.11 [0.88–1.41]	1.04 [0.82–1.33]
Highest quartile	1.32 [1.03–1.70]	1.31 [1.02–1.69]	1.20 [0.92–1.55]
% Single households^3^			
Lowest quartile	1.00	1.00	1.00
2^nd ^quartile	1.44 [1.14–1.80]	1.44 [1.15–1.81]	1.38 [1.10–1.75]
3^rd ^quartile	1.65 [1.33–2.05]	1.65 [1.33–2.06]	1.63 [1.31–2.04]
Highest quartile	1.44 [1.14–1.80]	1.38 [1.09–1.76]	1.35 [1.05–1.72]
% Single-parent families^4^			
Lowest quartile	1.00	1.00	1.00
2^nd ^quartile	1.08 [0.82–1.42]	1.06 [0.81–1.40]	1.03 [0.78–1.37]
3^rd ^quartile	1.43 [1.11–1.83]	1.43 [1.12–1.83]	1.37 [1.06–1.77]
Highest quartile	1.28 [1.00–1.65]	1.28 [1.00–1.64]	1.21 [0.94–1.57]

Model 1: Age adjusted

Model 2: Model 1 + family type adjusted

Model 3: Model 2 + SES + Education adjusted

^1 ^% employed of total labour force. Year 2001. Source: City of Helsinki Urban Facts.

^2 ^% manual workers of economically active residents. Year 2000. Source: Helsinki Region Statistics.

^3 ^% single households of all households. Year 2001. Helsinki Region Statistics

^4 ^% single-parent families of all families. Year 2001. Helsinki Region Statistics

Adjusting for the social composition of the areas decreased the variance between areas on average by about one fifth. We further assessed the role of composition by controlling for housing tenure, perceived financial difficulties and financial satisfaction (data not shown) but this did not affect the association between area characteristics and smoking.

## Discussion

There was considerable variation in female smoking by area, represented here by neighbourhoods in Helsinki. As middle-aged female smoking rates tend to be lower than in the general population and smoking is increasingly concentrated on the unemployed (male) population we expected not to find a small area effect in smoking in this population. Yet, we found that an additional risk of smoking can also be found among employed females residing in an area with a high level of unemployment, of single households and of single-parent families; adjusted smoking rates were between 21–35% higher in these areas.

At the individual level, the highest frequency of smoking was found among 45-year-old women, among those with the lowest level of education, single mothers and manual workers. However, even when taking into account the differences in these individual-level characteristics, we did not remove the association between smoking and area-level deprivation, which is in line with many earlier studies of representative samples of the adult population [26-35,45-47].

Our sample consisted of employees of the City of Helsinki and we limited our focus to women, as they represent the great majority (four fifths) of the employees of the City of Helsinki. In other words, even though the study setting can be seen to compromise the representativeness to some extent, it still allowed us to explore area variation in smoking in a fully employed population. In this respect, the magnitude of the area variation observed in this study is noteworthy as one might suspect this quite homogenous population to lack significant lifestyle differences. This is especially surprising given the low level of spatial differentiation in deprivation in Helsinki in comparison with e.g. London [48].

So far, the reasons for more prevalent smoking in socio-economically deprived and socially less cohesive areas are unclear. Obviously, a cross-sectional study does not allow causal inferences, which is why our interpretations must remain cautious. Yet, we may hypothesize that material smoking-related characteristics, such as the price of tobacco and the availability of cigarettes are unlikely to factor as they do not vary between urban neighbourhoods to any great extent. Instead, social norms and local cultural context may regulate smoking differently between areas. For example, more affluent areas may be less tolerant towards smoking in public places or in restaurants. In Finland, smoking outside designated areas in pubs and cafés has been banned since 2000. Furthermore, there may be more opportunities and less social control to smoke for example in the areas where single households form a major population group. As most Finns prefer not to smoke in their own home, then pubs, cafés and restaurants provide an environment for smoking. At the time of the survey, smoking was allowed in most public places. Currently smoking in pubs, cafés and restaurants is allowed only in designated areas and outdoors [49].

This study contributes to existing research by specifying the way a small local area may affect smoking. The area variables were conceptualised to measure social structure and social cohesion of the area. We found some tendency of the strongest area effects on smoking not among residents of the most, but the second most deprived areas, which can also probably be explained by the lack of a non-employed population in the sample. Their influence, particularly the influence of the unemployed population, on local smoking culture and other area-related factors is likely to be strongest on the most deprived areas where their presence is notable.

In part the associations may result from a local housing policy that blends population groups effectively. As council housing is available also in the more affluent neighbourhoods, the area-based segregation becomes less pronounced.

The study suggests that even though smoking may be to some extent contagious in areas with a high prevalence of smoking, there appears to be a distinctive mechanism as well. Thus, in the most deprived areas the employed females may want to distinguish themselves from the predominantly unemployed smoking male population by ceasing to smoke to a larger degree and they may be less likely to be in relationships with them. If this is correct, in the second most deprived area a similar social distance would not be reasonable as smokers would comprise a more heterogeneous group.

A similar process can be proposed with respect to low social cohesion. We expected cohesion to represent the quality of social life, which via norms would regulate the desirability of smoking. We did find evidence of increasing smoking with decreasing cohesion but again the area with second lowest cohesion featured the highest smoking rates. Clearly, the employed females are not affected by this type of normative climate to a similar degree in areas where smoking is, if not most desirable, at least, least un-desirable. Even though our data does not allow testing for the hypothesis we suspect this effect may be gender-specific so that the more permissive norms affect females less than males. This results from the fact that particularly in the most permissive areas male smoking is also most visible due to the public nature of smoking in Finland.

Methodologically, an analysis of an employed population controls for a number of potential confounders between area and smoking. For example, as smoking has been shown to associate with poor economic conditions [50] observed smoking differences between areas often reflect simply local variation in poverty. Our smoking measure was based on a simple self-reported question, which means that misclassification is possible. However, the smoking prevalence (22.8%) corresponds well to the national average of this age group (20.4%) taking into account the fact that rural females smoke less in these cohorts [51].

This study suggested a small significant area effect on smoking according to three variables measuring social structure and social cohesion of the area. The study probably produces quite a conservative estimate of the scale of the area effects. Even though the area differentiation in Helsinki city is considerable [37], some of the least deprived areas are located in other adjacent municipalities. Inclusion of data on residents of these other municipalities would probably have resulted in larger area effects as well.

Our failure to detect statistically significant random variance (between neighbourhoods) with coexisting statistically significant area-level fixed effects is evidently due to the lack of statistical power within our empirical model. As proposed by Diez-Roux [52] the test for random effects should not be decisive when the detection for any meaningful area-effect is performed in a comprehensive manner. In fact, we perceive our results to give support to the more liberal strategy for detecting neighbourhood effects put forward by Diez-Roux.

## Conclusion

Overall, our study showed social structural and social cohesive factors of the area to have a sizeable association with daily smoking among urban employed women. These effects were partly independent of individual socioeconomic characteristics and persisted after controlling for their compositional variation. In addition to a closer examination of the neighbourhood processes that induce smoking locally, the findings suggest that individual-based approaches to reducing smoking would benefit from community health promotion projects.

## Competing interests

The authors declare that they have no competing interests.

## Authors' contributions

SK conceived the study and the study design and drafted the manuscript. PS carried out the statistical analyses and contributed to their reporting. PM, OR and ML participated in designing the study and helped to draft the manuscript. All authors read and approved the final manuscript.

## Pre-publication history

The pre-publication history for this paper can be accessed here:


